# From Raffinose Family Oligosaccharides to Sucrose and Hexoses: Gene Expression Profiles Underlying Host-to-Nematode Carbon Delivery in *Cucumis sativus* Roots

**DOI:** 10.3389/fpls.2022.823382

**Published:** 2022-02-17

**Authors:** Xingyi Wang, Shihui Li, Xu Zhang, Lihong Gao, Yong-Ling Ruan, Yongqiang Tian, Si Ma

**Affiliations:** ^1^Beijing Key Laboratory of Growth and Developmental Regulation for Protected Vegetable Crops, College of Horticulture, China Agricultural University, Beijing, China; ^2^School of Environmental and Life Sciences and Australia-China Research Centre for Crop Improvement, The University of Newcastle, Newcastle, NSW, Australia

**Keywords:** *Cucumis sativus*, *Meloidogyne incognita*, sugar metabolism, RFOs, biotic stress

## Abstract

Root-knot nematodes (*Meloidogyne incognita*) induce specific feeding sites in cucumber roots where they absorb carbon nutrients from the host, thereby turning the feeding sites into a strong sink for assimilates. Nematode infection may alter host sugar metabolism in the roots of sucrose-transporting species. However, much less is known about the species translocating raffinose family oligosaccharides (RFOs), such as cucumber. To address this knowledge gap, the dynamics of RFOs and sucrose metabolisms, two major sugar-metabolism processes, in cucumber roots during nematode infection at transcription and protein levels were analyzed. In the nematode-infected root, the expressions of RFO-synthesis genes, *CsRS* (*Raffinose Synthase*) and *CsGolS1* (*Galactinol Synthase 1*), were upregulated at early stage, but were significantly decreased, along with *CsSTS* (*Stachyose Synthase*), at the late stage during nematode infection. By contrast, α-galactosidase hydrolyzed RFOs into sucrose and galactose, whose encoding genes was suppressed (*CsaGA2*) at early stage and then elevated (*CsaGA2*, *4*, and *CsAGA1*) at the late stage of nematode infection. Consistently, stachyose level was significantly increased by ∼2.5 times at the early stage but reduced at the late stage of infection in comparison with the uninfected roots, with a similar trend found for raffinose and galactinol. Moreover, the genes encoding sucrose synthase and cell wall invertase, which are responsible for sucrose degrading, were differentially expressed. In addition, sugar transporter, *CsSUT4*, was enhanced significantly after nematode infection at early stage but was suppressed at the late stage. Based on the observation and in connection with the information from literature, the RFOs play a role in the protection of roots during the initial stage of infection but could be used by nematode as C nutrients at the late stage.

## Introduction

The plant-parasitic nematodes are devastating pests causing billions of dollars in crop losses annually (Bozbuga et al., 2018). Among these nematodes, root-knot nematode (RKN) is a major sedentary endoparasitism, which is capable of inducing highly specific feeding cell systems in its host roots and damaging root development and function. This nematode could infect more than 5,000 plant species, causing approximately 30% yield loss by direct infestation and various degrees of indirect losses due to predisposition or breakdown of resistance to other root diseases (Castagnone-Sereno et al., 2013). So far, four kinds of RKNs comprising of *Meloidogyne incognita* (*M. incognita*), *Meloidogyne javanica* (*M. javanica*), *Meloidogyne arenaria* (*M. arenaria*), and *Meloidogyne hapla* (*M. hapla*) are considered as serious threats to crop production (Brinkman et al., 2015). Thus, understanding and preventing nematode parasitism are of importance in crop production.

After invading the host plant, the RKNs grow inside root and develop from juvenile to adult stages, the process of which remains for several weeks (Abad et al., 2008). Specifically, after egg hatching, the infective second-stage juvenile (J2) nematodes enter the vascular cylinder of root tips and move upward until near the elongation zone to form a feeding site (Perry and Moens, 2011). The vascular cells selected by J2 form several binucleate cells; these nuclei are further divided and lead to the formation of multinucleated giant cells (GCs). As the GCs develop, the neighboring cells proliferate and enlarge to form a characteristic hyperplastic structure called a root-knot or gall. After then, the RKNs become sedentary within the gall and feed on the nutrient channeled from GCs until life cycle completion. The GCs possess high metabolic activity and withdraw significant amounts of sugars and protein from the host to support the growth and reproduction of RKNs (Gommers and Dropkin, 1977; Bartlem et al., 2014; Escobar et al., 2015). The continuous withdrawal of nutrients by RKNs turns the GCs into strong nutrient sinks in the host roots. The nematode-induced galls in vascular cylinder and cortex cells of roots could impair water and mineral transport, inhibit shoot growth, and ultimately lead to irreversible yield loss.

The nematodes at feeding sites could use specific strategies to snatch host carbohydrates to fuel their growth (Slewinski, 2011). For most flowering plants, sucrose is the major end product of photosynthesis, sucrose metabolic enzymes, such as sucrose synthase (SUS), cell wall invertase (CWIN), and sugar transporters that may include hexose transporters (HTs) and sucrose transporters (H^+^-coupled sucrose transporter, SUT) were suggested to involved in the processes of phloem loading or unloading (Braun et al., 2014). In Arabidopsis, the genes encoding sucrose transporter (e.g., *AtSUC1* and *AtSUC2*), SUS, and CWINs were differently expressed in response to the infections of *Heterodera schaschtii* (*H. schaschtii*; cyst nematode) and *M. javanica* (Hofmann et al., 2007b,^2010^). In syncytia, the feeding site caused by *H. schaschtii*, no plasmodesmata were found between syncytia and surrounding cells at early stages, and sucrose was supplied to syncytia through H^+^-coupled sucrose transporter *AtSUC4* in Arabidopsis roots (Hofmann and Grundler, 2007; Hofmann et al., 2007b). After infecting *M. incognita*, the significant upregulation of *AtSUC4* suggested its role in the sucrose supply of GCs embedded in Arabidopsis root galls (Hofmann et al., 2009). Hammes et al. (2005) analyzed the expression of transporter genes in response to RKN infection and found significant changes in expression levels of at least 50 transporter genes from 18 different gene families, particularly sugar transporter genes including *AtSUC1*, *AtSTPs*, and *AtSFPs*. Differently, in rice root with an infection of *Meloidogyne graminicola*, the plasmodesmata-mediated, instead of sugar transporters-facilitated, sucrose transport plays key role in sugar supply to the GCs (Xu et al., 2021). Although the cyst nematodes and RKNs differ in terms of host selection and invasion mechanisms, they both withdraw nutrients from the host roots (Slewinski, 2011).

For some plant species, raffinose family oligosaccharides (RFOs) are the main sugars for phloem loading and translocation to sink organs. The RFOs involve in many growth and development processes including phloem translocation, storage of carbon in vegetative and reproductive tissues, and response to abiotic and biotic stresses (Taji et al., 2002; Pennycooke et al., 2003). In *Cucurbitaceae*, raffinose and stachyose are synthesized by raffinose synthase (RS) and stachyose synthase (STS) in the leaves and transported to sink organs *via* the phloem (Ma et al., 2019). During unloading, the RFOs are hydrolyzed into sucrose and galactose by α-galactosidase (α-Gal) and then undergo sucrose metabolic processes. As stated above, most of the previous studies focused on the sucrose-transport species (Hofmann et al., 2007a,b, 2009; Xu et al., 2021), however, in RFOs-transport species, there is still limited information regarding the response of sugar metabolism to nematode infection.

Cucumber (*Cucumis sativus* L.) is widely cultivated as an economical vegetable crop worldwide but could suffer from serious nematode infection, especially from a RKN, *M. incognita* (Kayani et al., 2017). Currently, cultivars or commercial rootstocks that are resistant to *M. incognita* are restricted to solanaceous crops such as tomato and pepper (Jacquet et al., 2005; Ros-Ibáñez et al., 2014) but barely reported in cucumber (Li et al., 2021). A major limitation to breed nematode-resistant cucumber varieties is insufficient knowledge on genes responding to, or mechanisms underlying, *M. incognita* infection in cucumber. Although sugar and its metabolism and transport in cucumber have been well-understood (Sui et al., 2018), their roles in and responses to nematode infection in cucumber are obscure. Considering they are the most important nutrient sources for nematode development (Escobar et al., 2015), they deserve more attention. There are large numbers of genes involved in sugar metabolism and transport in cucumber, including four *Galactinol Synthase (GolSs*), one *RS*, one *STS* (Ma et al., 2019), eight α-Gal genes (four acid α-gal genes, *CsaGAs*, and four alkaline α-gal genes, *CsAGAs*) (Sui et al., 2018), four *SUSs* (Wang et al., 2014), five *CWINs* (Hu et al., 2011), and three sugar transporters (*SUTs*) (Ma et al., 2019). However, there is still a lack of holistic understanding on whether any of these genes are regulated in response to nematode infection. To address this issue, in this study, the dynamics in sugar contents and gene expressions for RFO and sucrose metabolism and sugar transport were investigated in cucumber root with or without nematode infection. These findings provide theoretical basis for breeding for nematode-resistance cultivars of cucumber through manipulation of sugar withdrawal process.

## Materials and Methods

### Plant Materials and Growth Conditions

The cucumber (*Xintaimici*) seeds were sown in plastic pots containing sterilized sand:vermiculite [1:1, (v/v)]. The seedlings were grown in a growth chamber under day/night temperature of 28°C/18°C and during 16 h/8 h cycle. The stocks of *M. incognita* were maintained on the roots of soil-grown water spinach (*lpomoea aquatica Forsk* cv Liuye). The egg masses were collected from spinach root galls and placed in water at 28°C for 3 days. The preparasitic J2s (pre-J2s) were collected for cucumber root infection (Ding et al., 1998).

### Nematode Culture and Infection Assays

For infection assays, about 300 fresh pre-J2s were inoculated to invade the root of each cucumber seedling with two leaves. The samples were collected at 7–35 days postinoculation (dpi). At 7 dpi, the parasitic second juvenile stage nematode (J2s) are found in host plants. At 14 dpi, J2s and J3s are found and feeding sits initiate expansion. At 21 dpi, nematodes are in J3 stage and sexually differentiating, the female/male nematodes can be discerned. At 28 dpi, fourth-stage juveniles (J4) become adult nematodes. At 35 dpi, egg masses can be easily recognized. Full cucumber roots were sampled because the galls could not be easily discerned at 7 dpi, but the galls were sampled in the late stage of nematode infection (14, 21, 28, 35). The roots were speedily washed in 0.1% diethyl pyrocarbonate (DEPC) water, then the infected galls and corresponding uninfected lateral roots were immediately frozen in liquid nitrogen, and stored in –80°C until use. For RNA extraction and enzyme activity analysis, 100 mg of fresh roots or galls were collected in each sample, and 500 mg of fresh roots or galls were collected for detecting sugar content.

### Nematode Staining Tests

For observing the developmental stages of nematode, the cucumber roots were infected by pre-J2s *M. incognita* and then stained by using acid fuchsin according to Bybd et al. (1983) with some modifications. First, the cucumber roots were soaked in 1.5% NaClO_4_ for 4 min and rinsed with tap water for 45 s. Then, they were soaked for 15 min with distilled water to remove residual NaClO_4_ and dried. The roots were placed in 30 ml distilled water and 1 ml of acid-fuchsin solution was added and boiled for 30 s. Subsequently, the stained roots were washed with running water and transferred to acidic glycerin with heating to fade. Finally, the roots were stored overnight in acidic glycerin for detection of nematodes.

### Carbohydrate Extraction

The extraction of soluble sugar was based on the method of Ma et al. (2019). The roots or galls frozen in liquid nitrogen were grounded into powder in a mortar with 5 ml of 80% (v/v) ethanol and 80°C water bath for 30 min, centrifuged at 4,000 × g for 10 min (H2050R, Xiangyi Centrifuge Instrument Co., Ltd., China), and then the supernatant was collected. The precipitate after centrifugation was repeated three times for extraction. All the supernatants collected from the three extractions were vacuum dried. After resuspending the dried sample in 1 ml deionized water, the sample was filtered using the filter membrane with a diameter of 0.45 μm and detected by high performance liquid chromatography.

### RNA Isolation and Quantitative Real-Time PCR

The total RNA was extracted from the lateral roots and galls using RNeasy Plant kit (Huayueyang, Beijing, China) according to the manufacturer’s protocol. For each case, 1 μg RNA was used for cDNA synthesis using the Faster Quant RT kit (Qiagen, Beijing, China). The cDNA samples were used as templates for RT-qPCR analysis. Quantitative RT-PCR was performed in 384 well plates with a 7,500 real-time (RT) PCR System from Applied Biosystems using SYBR Green according to the manufacturer’s protocol (Takara, Japan). All of the samples were tested in five biological replicates with two technical replicates for each reaction. *CsUBQ* (Csa4G089780) and *CsEF1*α (Csa2G139820) were employed as internal reference genes. The results were determined by using the 2^–△△CT^ method (Schmittgen and Livak, 2008). The primers and gene accession data are listed in Supplementary Tables 1, 2, respectively.

### Enzyme Activity Analysis

The samples (0.5 g) of lateral roots and galls were grounded in a mortar containing liquid nitrogen. The SUS was measured using Sucrose Synthase (degradative direction; SUS) Activity Assay Kit (Solarbio, BC4315, Beijing, China). The SUS catalyzes sucrose degradation in the presence of uracil diphosphate (UDP) to produce fructose and UDP-glucose at the pH 5.5. The reactions were proceeded for 20 min at 30°C and were terminated by 95°C for 5 min. The fructose reacts with 3, 5-dinitrosalicylic acid to produce brown-red substance with characteristic absorption peak at 540 nm. The SUS activity can be calculated by measuring the change of absorption value at 540 nm. Briefly, the evenly ground samples were added with 1 ml extraction buffer and centrifuged at 8,000 × g for 10 min at 4°C. The supernatant was collected and placed on ice for enzyme activity measurement according to the instruction of kit and Wang et al. (2014) with some changes.

The CWIN was extracted and determined using Cell Wall-Bound Acid Invertase Activity Assay Kit (Solarbio, BC4325, Beijing, China) according to the instruction of kit and Palmer et al. (2015) with some modifications. The CWIN catalyzes sucrose to produce glucose and fructose, which react with 3, 5-dinitrosalicylic acid to produce brown-red substance with characteristic absorption peak at 540 nm (Thermo Fisher Scientific Oy, Ratastie 2, FI-01620 Vantaa, Finland). Mainly, the grounded samples were added with 1 ml extraction buffer I and centrifuged at 12,000 × g for 10 min at 4°C (H2050R, Xiangyi Centrifuge Instrument Co., Ltd., China). The supernatant was discarded and the sediment was collected and added with 1 ml distilled water before fully mixed. Once again the sediment was retained by centrifugation at 12,000 × g for 10 min. Then, the precipitate was added with l ml extraction buffer II and fully blended. The sample was incubated at 4°C for 15 h and centrifuged at 12,000 × g for 10 min at 4°C. The supernatant was collected and placed on ice for enzyme activity measurement with Thermo Scientific Microplate Reader.

The α-galactosidase activity was determined by using the α-Galactosidase Activity Assay Kit (Solarbio, BC2575, Beijing, China) following the instruction of kit. The α-galactosidase decomposes p-nitrobenzene-α-D-galactoside to p-nitrophenol, the latter has the maximum absorption peak at 400 nm. The activity of α-galactosidase was calculated by measuring the increasing rate of the absorption value. Mainly, the homogenous sample was added with 1 ml extraction buffer by centrifuging at 15,000 × g for 20 min at 4°C. The supernatant was collected for enzyme activity assay using Thermo Scientific Microplate Reader.

In all the enzyme activity assays, the protein concentration was measured using the Bradford Method Protein Quantitative Kit (Huayueyang, PP0202, Beijing, China).

### Statistical Analysis

For RNA extraction and enzyme activity analysis, six plants were pooled as one biological replicate with 5 replicates and three technique repeats each replicate used in each experiment. For detecting the sugar content, six plants were pooled as one biological replicate with 3 replicates. The experimental data were expressed as mean ± SE. The experimental data were analyzed using statistical product and service solutions (SPSS) v. 30.0 (SPSS Inc.). The data significant differences were analyzed using a two-tailed Student’s *t*-test, values with *p* < 0.05, *p* < 0.01, or *p* < 0.001 were considered statistically significant.

## Results

### Development of *M. incognita* in Cucumber Roots

**FIGURE 1:**
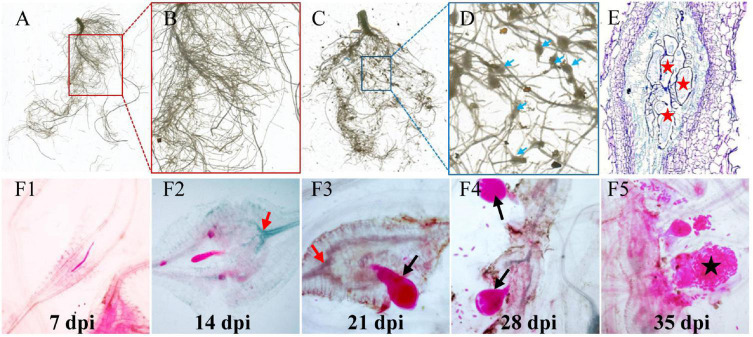
Infection of *M. incognita* to cucumber roots. **(A)** Control cucumber roots without nematode infection. **(C)** Galls (blue arrows) are induced in roots by *M. incognita*. **(B,D)** Are the close-up views of sections marked in **(A)** (red) and **(C)** (blue) regions. Blue arrows in **(D)** indicate root knots or galls induced by nematodes. **(E)** The anatomic structure of a gall. Red pentagrams indicate the giant cells (GCs). **(F1–F5)** RKN life cycle inside cucumber roots, J2 **(F1)**-J3 (**F2**)-J4 **(F3,F4)**-eggs **(F5)**. Black arrows indicate the female nematodes with a pear-shaped body. Black pentagram indicates the eggs produced by adult nematodes.

To understand the RKN life cycle inside cucumber roots for the selection of the right stage of nematode development for analyses, *M. incognita*-induced galls were observed in *Cucumis sativus* (Figure 1). Visible galls were observed in cucumber root systems at 7–35 days postinoculation (dpi) by *M. incognita* (Figures 1A–D). To investigate the intercellular states of nematodes along different timepoints in infected cucumber roots, histological experiments were conducted. At 7 dpi, the feeding sites (GCs) were formed in vascular cylinder, with later continuous expanding at 14 dpi (Figure 1E), and a high increase of nematodes (juveniles, adults, and eggs) was observed along different stages of infected roots at 21, 28, and 35 dpi (Figures 1F1–F5). Based on the stage of nematode development, the galls at typical timepoints (0, 7, 14, 21, 28, and 35) were sampled for further study.

### Soluble Sugar Dynamics in Nematode-Infected Cucumber Roots

**FIGURE 2:**
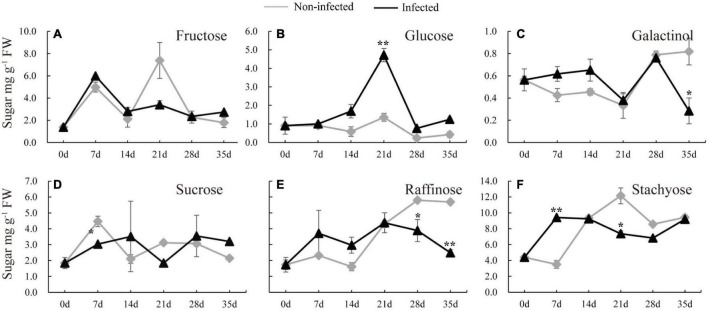
Changes of carbohydrate content in infected and non-infected cucumber roots by *M. incognita*. **(A–F)** Are the content of fructose, glucose, galactinol, sucrose, raffinose and stachyose in cucumber roots, respectively. Error bars represent ± SE (*n* = 6). Student’s *t*-test is analyzed between infected and non-infected roots, *P < 0.05; **P < 0.01.

To understand how nematode infection may affect sugar status in the root, the soluble sugars were measured in the infected cucumber roots (Figure 2). Five timepoints (7, 14, 21, 28, and 35 days) selected from infected and non-infected roots were chosen. Compared with non-infected roots, the stachyose level was induced and sucrose was decreased at 7 dpi (Figures 2D,F). In the late stage of nematode infection, glucose, but not fructose, was significantly induced at 21 dpi, however, galactinol was decreased at 35 dpi, raffinose was decreased at 28 and 35 dpi, and stachyose was decreased at 21 dpi in infected roots compared with non-infected roots (Figures 2B–F).

### Responses of *CsRS*, *CsSTS*, and *CsGolS1* in Nematode-Infected Roots

**FIGURE 3:**
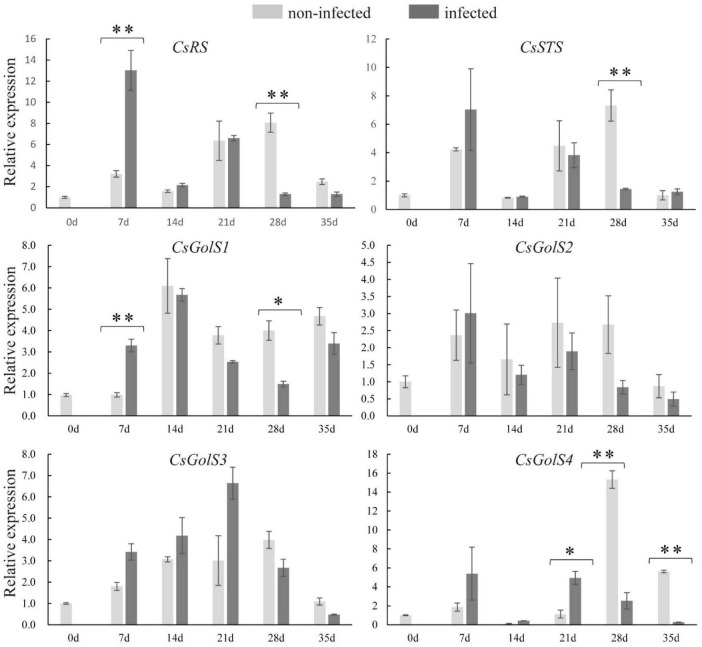
Expression levels of *CsRS*, *CsSTS*, *CsGolSs* in infected and non-infected cucumber roots by *M. incognita*. *CsRS*, raffinose synthase gene; *CsSTS*, stachyose synthase gene; *CsGolS1–4*, galactinol synthase genes, respectively. Error bars represent ± SE (*n* = 5). Student’s *t*-test is analyzed between infected and non-infected roots, *P < 0.05; ^**^P < 0.01.

To examine whether RFOs metabolism responded to nematode infection, qRT-PCR was performed to determine the expressions of *CsRS*, *CsSTS*, and *CsGolSs*. Compared with the non-infected roots, the transcript levels of *CsRS* and *CsGolS1* were enhanced at 7 dpi (Figure 3). However, their mRNA levels, together with that of *CsSTS*, were suppressed at 28 dpi in infected cucumber roots, with no difference detected at 14 dpi (Figure 3). This result was consistent with the finding that RFOs (galactinol, raffinose, and stachyose) were decreased at the late stage of nematode infection (Figure 2). The expression of *CsGolS4* was increased at 21 dpi, but then decreased at 28 and 35 dpi, in the infected roots, however, its expression was weak (qPCR *Ct* value was ∼32). By comparison, the expressions of *CsGolS2* and *CsGolS3* were not affected by nematode infection (Figure 3). Thus, *CsGolS1* dominated most of the GolS transcripts in cucumber roots under nematode infection.

**FIGURE 4:**
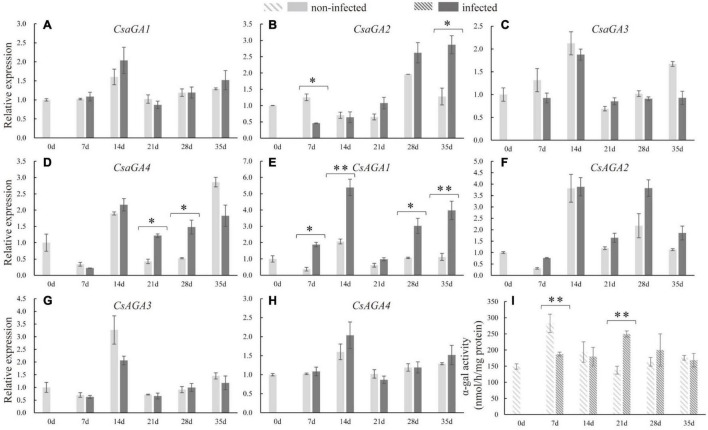
Expression levels of α-galactosidase genes and α-galactosidase activity analysis in infected and non-infected cucumber roots by *M. incognita.* **(A–D)** Are acid α-galactosidase genes, *CsaGA1* **(A)**, *CsaGA2* **(B)**, *CsaGA3* **(C)** and *CsaGA4* **(D)**. **(E–H)** Are alkline α-galactosidase genes, *CsAGA1* **(E)**, *CsAGA2* **(F)**, *CsAGA3* **(G)** and *CsAGA4* **(H)**. **(I)** Is α-galactosidase activity analysis. Error bars represent ± SE (*n* = 5). Student’s *t*-test is analyzed between infected and non-infected roots, *P < 0.05; ^**^P < 0.01.

The expression levels of RFOs degradation-related genes (*CsaGAs* and *CsAGAs*) encoding α-galactosidase (α-gal) were also examined. The expression of *CsAGA1* was stimulated at 7, 14, 28, and 35 dpi, the expression of *CsaGA4* was enhanced at 21 and 28 dpi, whereas the expression of *CsaGA2* was suppressed at 7 dpi but then induced at 35 dpi in the infected roots compared with the non-infected roots (Figures 4A–D). However, *CsaGA1*, *3*, and *CsAGA2–4* did not respond to nematode infection (Figures 4E–H). The enzyme assay revealed that α-gal activity was significantly suppressed at 7 dpi but enhanced at 21 dpi (Figure 4I). Overall, these results showed that RFO metabolism seemed to be associated with the infection by nematodes.

### Sucrose Synthase and Cell Wall Invertase Gene Expressions During Nematode Infection

**FIGURE 5:**
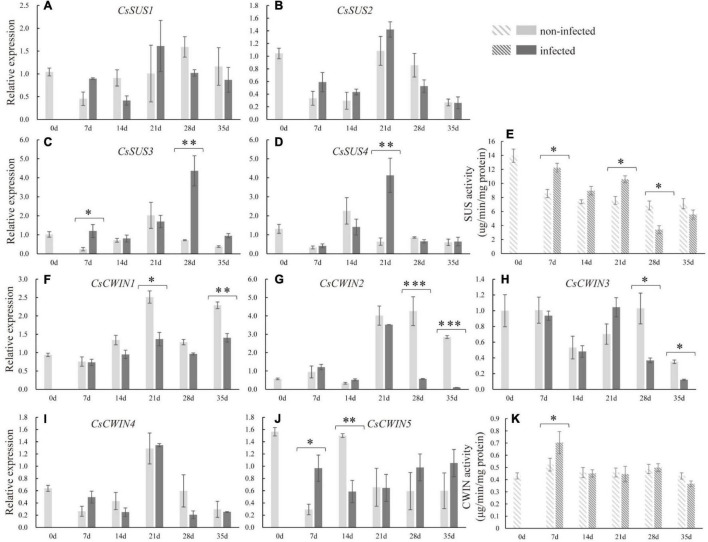
Expression levels of sucrose synthase genes (*CsSUS1–4*) **(A–D)**, cell wall invertase genes (*CsCWIN1–5*) **(F–J)**, and the enzyme activity analysis of CsSUS **(E)** and CWIN **(K)** in infected and non-infected cucumber roots by *M. incognita*. Error bars represent ± SE (*n* = 5). Student’s *t*-test is analyzed between infected and non-infected roots, *P < 0.05; ^**^P < 0.01; ^***^P < 0.001.

To explore the molecular basis underlying sucrose metabolism and transport upon infection, related gene expression levels and enzyme activities in infected cucumber roots were measured. At 7 dpi, the expression of *CsSUS3* was stimulated. At the late stage of nematode infection, the expressions of *CsSUS3* at 28 dpi and *CsSUS4* at 21 dpi were enhanced in infected roots (Figures 5C,D). Regarding the expression of *CsSPSs* in infected roots, *CsSPS1* and *CsSPS2* were suppressed in infected roots compared with non-infected roots at 7 and 35 dpi, respectively (Supplementary Figure 1). Moreover, at the early stage of nematode infection (at 7 dpi), the expression of *CsCWIN5* was increased in infected roots. However, the expression of *CsCWIN1* at 21 and 35 dpi, *CsCWIN2* and *3* at 28 and 35 dpi, and *CsCWIN5* at 14 dpi were suppressed in infected roots (Figures 5F–J). By comparison, nematode infection did not affect the expression of *CsSUS1*, *CsSUS2*, *CsSPS4*, and *CsCWIN4* (Figures 5A,B,H and Supplementary Figure 1). The enzyme activity assay revealed that CsSUS was enhanced at 7, 14, and 21 dpi, but decreased at 28 dpi, while CsCWIN was increased at 7 dpi (Figures 5E,K).

### Responses of Sugar Transporter Genes in Nematode-Infected Roots

**FIGURE 6:**
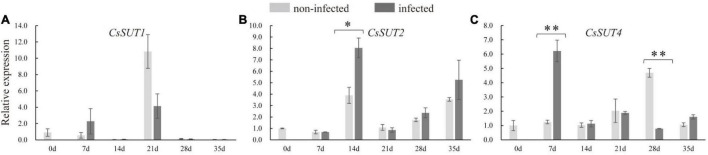
Expression levels of sucrose transporter genes (*CsSUTs*) **(A–C)** in infected and non-infected cucumber roots by *M. incognita*. Error bars represent ± SE (*n* = 5). Student’s *t*-test is analyzed between infected and non-infected roots, *P < 0.05; ^**^P < 0.01.

In order to identify whether sugar transporter genes respond to nematode infection in cucumber, the expression of *CsSUTs* and *CsHTs* were examined. The qRT-PCR analysis revealed that the expression of *CsSUT4* was induced at 7 dpi and suppressed at 28 dpi in infected roots (Figure 6), and *CsSUT2* expression was increased at 14 dpi in infected roots; however, the expression of *CsSUT1* did not respond to nematode infection. The HTs were also involved in sugar partitioning processes. Here, the expression of *CsHT1* and *CsHT4* was downregulated at 21 and 14 dpi in infected roots, respectively (Supplementary Figure 2). By comparison, the expression of *CsSUT1* and *CsHT3* showed no change in infected roots by nematode infection (Figure 6 and Supplementary Figure 2).

## Discussion

The biotrophic parasites could alter carbohydrate metabolism in infected host plants to acquire sugars to fuel their growth (Hofmann et al., 2007a). Previous studies have described the changes of sugar metabolism and import into syncytia of sucrose-transporting species in response to *H. schachtii* infection (Hammes et al., 2005; Hofmann et al., 2007a,b; Cabello et al., 2014). However, it remains elusive how sucrose or RFOs are imported into and utilized within the *M. incognita*-induced GCs in RFOs-transported species.

### Dual Roles of Raffinose Family Oligosaccharides in Nematode Resistance and Feeding

The first step of nematode infection in host is to break the physical barrier, cell wall, for entering the host’s vascular cylinder. Plethora studies suggested that the intracellular accumulation of RFOs metabolic enzymes are closely associated with the response to environmental stresses no matter in sucrose- or RFOs-transported species (Klotke et al., 2004; Peters and Keller, 2009; Ma et al., 2021). Previous studies have supported that RFOs could be inserted into lipid headgroups of membrane bilayer and stabilize membrane when under stress, and RFOs could interact with ROS signaling pathway and act as a free radical scavenger (Hincha et al., 2003; Nishizawa et al., 2008). In Arabidopsis, a sucrose-transported species, the sugar was transported into feeding sites induced by RKNs/cyst nematodes and stored as starch for carbohydrate need of nematodes (Hofmann et al., 2007a). In addition, previous studies have revealed that plants produce osmotic substances, such as sucrose and RFOs, to resist stress (Zulfiqar et al., 2020). In previous study, after being infected by cyst nematode, raffinose and galactinol were increased, whereas stachyose was not detected in Arabidopsis roots over the course of development (from 5 to 15 dpi) (Hofmann et al., 2010). In infected cucumber, stachyose content was increased in roots at early stage (7 dpi), with a similar pattern observed in raffinose and galactinol (Figure 2), and the expression of *CsRS* was significantly induced at 7 dpi, it was possibly that synthesized raffinose and galactinol may be further converted to stachyose by STS in the infected cucumber roots. This result showed that stachyose plays a key role at early stage of nematode infection in cucumber.

In the current study, at the late stage of nematode infection, stachyose, raffinose, and galactinol contents were decreased, consistent with the downregulated transcript level of *CsSTS*, *CsRS*, and *CsGolS1* and increased α-gal activity in infected cucumber roots (Figures 2, 3), whereas glucose was significantly accumulated in infected roots at 21 dpi (Figure 2). These results suggested that RFOs were degraded by α-galactosidase likely for providing sucrose and hexose at the late stage for nematode development, which was also supported by the induced α-gal enzyme activity at 21 dpi (Figure 4). The infected cucumber roots accumulated RFOs at the early stage of nematode infection (Figure 2). We speculated that the promoted RFOs might be relevant to resist the nematode infection at the early stage. However, the RFOs tended to be cleaved for supplying sugar for nematode spawning at the late stage of nematode infection. Thus, we proposed that the RFOs could play dual roles in the host-nematode interactions in cucumber, including resistance at the early stage and fueling nutrient at the late stage. This finding supports the possibility to breed nematode-resistant cultivars through manipulating RFO metabolism in *Cucurbitaceae* plants.

### Different Responses of *CsSUSs* and *CsCWINs* After Nematode Infection

In this study, the glucose was increased significantly at the late stage of nematode infection, but the RFOs content was decreased (Figures 2B,E). This finding suggests that, in cucumber, the glucose was the major induced sugar in galls and thus may play important role in fueling nematode development. Consistently, the expressions of *CsSUS3* and -*4* were enhanced in cucumber roots after infecting nematode, and the CsSUS activity was also promoted at 7 and 21 dpi (Figures 5A–D). Interestingly, the genes encoding CWIN, *CsCWIN5* was induced at the early stage, while *CsCWIN1*, *-2*, and -*3* were decreased at the late stage, in infected root (Figures 5F–J). As CsSUS was the only promoted sucrose-degrading enzyme at the late stage, the increased glucose at 21 dpi was very likely generated from sucrose degradation by SUS. The CsCWIN activity was induced only at the early stage, but not at the late stage, of nematode infection (Figure 5), which was in accordance with the enhanced expression of *CsCWIN5* (Figure 5J). The transcriptional level of *CsCWINs* and enzyme activity of CsCWIN showed different change trends at the late stage of nematode infection, and this was possibly caused by posttranscriptional regulation or posttranslational regulation (Jin et al., 2009). It had been proposed that invertase was posttranscriptionally regulated when plants suffered from biotic stresses (Bonfig et al., 2010). In this study, the result of increased CsCWIN and CsSUS activity in infected cucumber roots was similar with previous findings that SUS and CWIN activity was induced in syncytia (3 dpi) (Cabello et al., 2014). Together with the previous findings on *SUS* and *CWIN* mutants of Arabidopsis demonstrating less resistance to *H. schaschtii* (Cabello et al., 2014), our finding suggests that in cucumber, a RFO-transporting species, CsCWIN and CsSUS may relate to nematode resistance. In this study, glucose was promoted at the late stage (Figure 2), which may be contributed by (i) sucrose degradation by enzymes of SUS and CWIN and (ii) galactose metabolism after RFO being degraded into galactose by α-gal. This statement is supported by the promoted SUS and CWIN and decreased RFO contents at the later stage of nematode infection (Figures 2, 5). The roles of SUS and CWIN in nematode infection and resistance deserve further investigation, and evidence regarding functional identification, protein localization, and other methodologies may provide more insight into the parasitism of nematode in cucumber.

**FIGURE 7:**
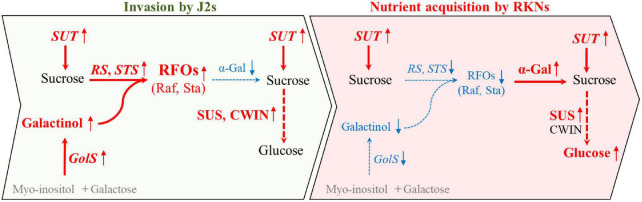
A model describing the distinct responses of sugar contents and the expression of metabolic genes at early (invasion by J2s) and later (nutrient acquisition by RKNs) stages during infection. During the invasion of nematode in cucumber roots, *CsGolS* was induced and galactinol was synthesized with myo-inositol and galactose. Further, RFOs may be largely synthesized by CsRS and CsSTS from galactinol and sucrose which was transported by SUT at early stage, possibly for resisting the infection of nematode. Afterward, upon nematode development, RFOs may be cleaved to sucrose and galactose by α-gal. Furthermore, sucrose was degraded by SUS encoded into hexoses at early and late stage of nematode infection, providing energy and carbon to supply nematode development. Red font and blue font indicate increase and decrease after nematode infection compare with non-infection, respectively. Black font imply no differences between infected and non-infected by nematode. Gray parts have not been detected in this study.

Collectively, we propose that the RFOs were largely synthesized by *CsRS*, *CsSTS*, and *CsGolS1* at the early stage for resisting the infection of nematode. Afterward, upon the nematode has infected and proceed to develop, the RFOs were cleaved by α-gal encoded by *CsAGA1* and *CsaGA4* to sucrose and galactose. The sucrose was further degraded by CsSUS encoded by *CsSUS3*, *-4* into hexoses, providing energy and carbon to supply nematode development (Figure 7). These findings identified the candidate genes involved in nematode infection for further studies and provided new insight into the sugar metabolic dynamics in cucumber roots infected by *M. incognita*.

## Data Availability Statement

The datasets presented in this study can be found in online repositories. The names of the repository/repositories and accession number(s) can be found in the article/Supplementary Material.

## Author Contributions

SM and YT conceived the project and designed the experiments. XW, SL, and XZ conducted the experiments and collected the data. SM, XW, and SL analyzed the data and wrote the manuscript with inputs from Y-LR and LG. All authors contributed to the article and approved the submitted version.

## Conflict of Interest

The authors declare that the research was conducted in the absence of any commercial or financial relationships that could be construed as a potential conflict of interest.

## Publisher’s Note

All claims expressed in this article are solely those of the authors and do not necessarily represent those of their affiliated organizations, or those of the publisher, the editors and the reviewers. Any product that may be evaluated in this article, or claim that may be made by its manufacturer, is not guaranteed or endorsed by the publisher.
