# Supplementary material for: From Raffinose Family Oligosaccharides to Sucrose and Hexoses: Gene Expression Profiles Underlying Host-to-Nematode Carbon Delivery in Cucumis sativus Roots
Source: Front Plant Sci. 2022 Feb 17;13:823382. doi: 10.3389/fpls.2022.823382 (PMC8892300; doi:10.3389/fpls.2022.823382)
Supplement: Supplementary file 1 [file Data_Sheet_1.PDF]

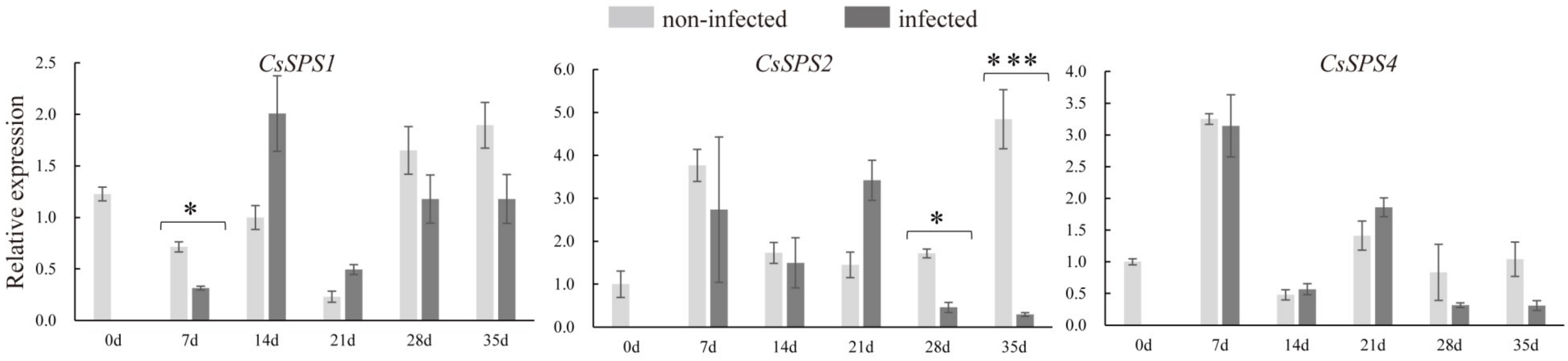

Supplemental figure 1. Expression levels of sucrose phosphorate synthase genes (*CsSPS1-3*) in infected and non-infected cucumber roots by *Meloidogyne incognita*. Error bars represent ± SE (n = 5). Student's *t*-test is analyzed between infected and noninfected roots, * $P < 0.05$; ** $P < 0.01$.

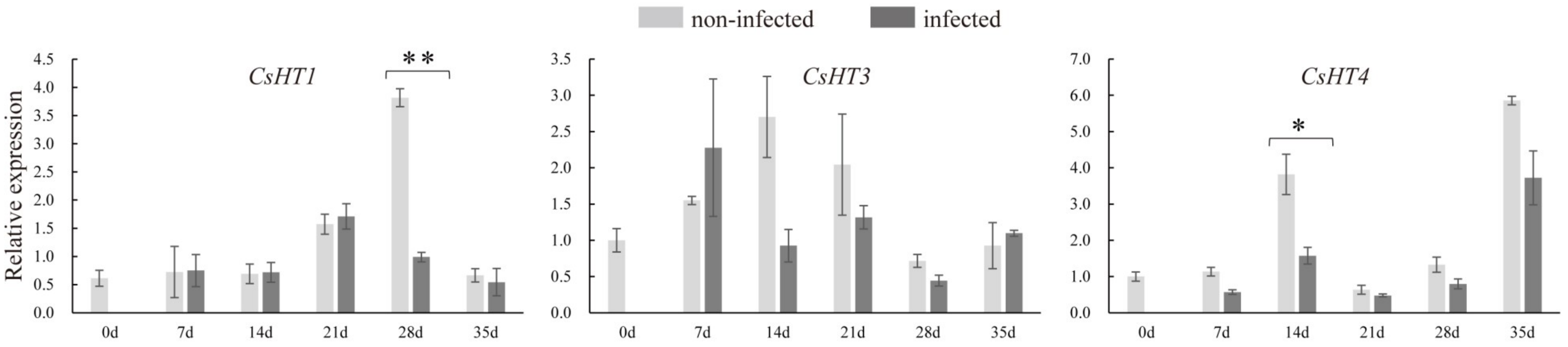

Supplemental figure 2. Expression levels of hexose transporter genes (*CsHTs*) in infected and non-infected cucumber roots by *Meloidogyne incognita*. Error bars represent ± SE (n = 5). Student's *t*-test is analyzed between infected and noninfected roots, * $P < 0.05$; ** $P < 0.01$.
