# Supplementary material for: From Raffinose Family Oligosaccharides to Sucrose and Hexoses: Gene Expression Profiles Underlying Host-to-Nematode Carbon Delivery in Cucumis sativus Roots
Source: Front Plant Sci. 2022 Feb 17;13:823382. doi: 10.3389/fpls.2022.823382 (PMC8892300; doi:10.3389/fpls.2022.823382)
Supplement: Supplementary file 2 [file Table_1.pdf]

Supplemental Table 1 qRT-PCR primers used in this study

genes	sequence of primers (5'-3')	genes	sequence of primers (5'-3')
**CsSPS1**	ACACGTTTGCCAAGAATCAGTT<br>CAAGCCTTCTGAATTAGTTGGAGTC	**CsAGA2**	GAAGATTACTGTCAACGATGGC<br>AAACAACAAAGGAAGCGAGTAC
**CsSPS2**	GTGCCACCGCCATTGAAGAACAGAA<br>GAGCTCCGTTCACGAGTGTTGCG	**CsAGA3**	GTTTATGTCAGTGATGCACCAG<br>GAGAATAAACAGTCCCGTGTTG
**CsSPS4**	AAGCAAAAAACAAGGAGGAAAGCAC<br>CAACTTCCCTTTCTTTTGTTTGTCTT	**CsAGA4**	AAACATGAAACAGCTGAGTTCC<br>GAAATCATGATTCCCAGGCTTG
**CsGolS1**	AGATGAATCTTTGGATTACCAAAATG<br>TTGACAACTTCAGCCTCGGAC	**CsaGA1**	TGGTGGTGTTTGTTTTAGCG<br>CCAGAGCATCAGCGGTTT
**CsGolS2**	AGTGATCCTTCTTTGGACTACAAGCC<br>GTGACGAAGTGAACAGGTCCAGC	**CsaGA3**	ATGCTGGTCTTTTCACTTGTCA<br>GGTGGGTATCGCTTTATTGGT
**CsGolS3**	GACGAAACCTTGGACTACATCAACTAT<br>GGCAGTGTGGCCGTAATGAAC	**CsaGA4**	GTTCCTGGGGCATACACTG<br>CAGTGTATGCCCCAGGAAC
**CsGolS4**	TCGCTCGATTTGAAGGAAGTGG<br>GAACATAGGCAATATTGGGCTCAG	**CsaGA2**	ATGGTCTCGCTCTAACTCCTC<br>GATACCTTTCTTTTGGGCTTG
**CsRS**	TTCGGTTTATGAGTATTTTCAGGTTTA<br>GACAAAGTCACTATCCCCAGG	**CsSUS1**	TCGTCATCTGTCATCGGTTA<br>CGAATAGGGTGTACTGGGCAGCAACTT
**CsSTS**	CAACTGTCAAGGAGCAGGGTGGG<br>AGCGGTTCGGATTTTGGGGT	**CsSUS2**	AGAAGATCCCGTAACCAAATGAAGA<br>AAAACATGGCTTCTTTCACG
**CsSUT1**	CGTGGTTACAAAGGTTGCTGAG<br>GAGAGGGGTAAACAGTGAATC	**CsSUS3**	AGAGACCGAGAAAAGGCTAACT<br>AACTAACTCCCTAAGACGATCG
**CsSUT2**	TTCTAACTCCCTATATTCAGACGCTC<br>GCAATGCTCCTTTGTATCTCCTAA	**CsSUS4**	CATTTTTCTTGTCAATTTACTGCTG<br>GCCCGACACGAAACGAC
**CsSUT4**	GTCCTCCCTGGTTGTAGAAGAAAG<br>CTCTACCCATCCAATCAGTATCAAAG	**CsCWIN1**	CCAACCGAACCTTATGAA<br>CAGCCAAGCAGTAGTAGGG
**CsHT1**	CATCATGCAACCAAGATACAGG<br>AGACATAAGTGAAGCACTGTCA	**CsCWIN2**	AGATTTCTTCCCTGTGGC<br>ATAAACGTCGTGCTTGGT
**CsHT3**	CTTTCCCCGCTTCAATCTTAAC<br>GATGGATTTAGCAATCTCGCTG	**CsCWIN3**	CTGTCATCGGCAGCAAGA<br>TAGCGGCAGAATGTAAAG
**CsHT4**	CGGCGGCGTCTTTGTATTC<br>GCCTTTTGATAGCTCCTTTCCTTG	**CsCWIN4**	GTCCTGGGTTTGACTTTA<br>TCCAGACCATCCCTTATT
**CsAGA1**	GTCTTCATCCTATGGCCGAATA<br>ATAGAACCATCATGGAGCACAA	**CsCWIN5**	CCGTCGCAGGCAGATGTT<br>CTGCTTTGGTCGCTACAC
**CsUBQ**	CACCAAGCCCAAGAAGATC<br>TAAACCTAATCACCACCAGC	**CsEF1α**	ACTGTGCTGTCCTCATTATTG<br>AGGGTGAAAGCAAGAAGAGC
