# Supplementary material for: From Raffinose Family Oligosaccharides to Sucrose and Hexoses: Gene Expression Profiles Underlying Host-to-Nematode Carbon Delivery in Cucumis sativus Roots
Source: Front Plant Sci. 2022 Feb 17;13:823382. doi: 10.3389/fpls.2022.823382 (PMC8892300; doi:10.3389/fpls.2022.823382)
Supplement: Supplementary file 3 [file Table_2.pdf]

Supplemental Table 2 Gene accession in this study

Gene	Accession	Gene	Accession
CsSPS1	CsaV3_1G027950.1	*CsAGA2*	CsaV3_4G011820.1
CsSPS2	CsaV3_2G011150.1	*CsAGA3*	CsaV3_1G026710.1
CsSPS4	CsaV3_2G033300.1	*CsAGA4*	CsaV3_1G000950.1
CsGolS1	CsaV3_6G000050.1	*CsaGA1*	CsaV3_5G029180.1
CsGolS2	CsaV3_3G031720.1	*CsaGA3*	CsaV3_5G010860.1
CsGolS3	CsaV3_1G015730.1	*CsaGA4*	CsaV3_2G025010.1
CsGolS4	CsaV3_5G031520.1	*CsaGA2*	CsaV3_5G029190.1
CsRS	CsaV3_3G042690.1	*CsSUS1*	CsaV3_4G000970.1
CsSTS	CsaV3_7G030090.1	*CsSUS2*	CsaV3_1G005170.1
CsSUT1	CsaV3_2G010720.1	*CsSUS3*	CsaV3_1G041400.1
CsSUT2	CsaV3_5G025170.1	*CsSUS4*	CsaV3_5G020420.1
CsSUT4	CsaV3_2G030550.1	*CsCWIN1*	CsaV3_4G035700.1
CsHT1	CsaV3_2G010410.1	*CsCWIN2*	CsaV3_7G034730.1
CsHT3	CsaV3_4G004110.1	*CsCWIN3*	CsaV3_3G037440.1
CsHT4	CsaV3_2G002050.1	*CsCWIN4*	CsaV3_2G025540.1
CsAGA1	CsaV3_4G033560.1	*CsCWIN5*	CsaV3_7G034730.1
CsUBQ	CsaV3_2G010720.1	*CsEF1-α*	CsaV3_2G011610.1
